# Coinfection with influenza A(H1N1)pdm09 and dengue virus in fatal cases

**DOI:** 10.1590/0074-02760160140

**Published:** 2016-09-01

**Authors:** Anne Carolinne Bezerra Perdigão, Izabel Letícia Cavalcante Ramalho, Maria Izabel Florindo Guedes, Deborah Nunes Melo Braga, Luciano Pamplona Góes Cavalcanti, Maria Elisabeth Lisboa de Melo, Rafael Montenegro de Carvalho Araújo, Elza Gadelha Lima, Luciene Alexandre Bié da Silva, Lia de Carvalho Araújo, Fernanda Montenegro de Carvalho Araújo

**Affiliations:** 1 Laboratório Central de Saúde Pública, Setor de Virologia, Fortaleza, CE, Brasil; 2 Universidade Estadual do Ceará, Rede Nordeste de Biotecnologia, Fortaleza, CE, Brasil; 3 Centro Universitário Christus, Fortaleza, CE, Brasil; 4 Universidade de Fortaleza, Fortaleza, CE, Brasil; 5 Universidade Federal do Ceará, Fortaleza, CE, Brasil; 6 Instituto de Prevenção de Câncer, Fortaleza, CE, Brasil; 7 Hospital Santa Casa de Misericórdia, Fortaleza, CE, Brasil

**Keywords:** dengue, fatal case, influenza A(H1N1)pdm09

## Abstract

We report on four patients with fatal influenza A(H1N1)pdm09 and dengue virus coinfections. Clinical, necropsy and histopathologic findings presented in all cases were characteristic of influenza-dengue coinfections, and all were laboratory-confirmed for both infections. The possibility of influenza and dengue coinfection should be considered in locations where these two viruses’ epidemic periods coincide to avoid fatal outcomes. Dengue is a mosquito-borne viral infection caused by one of the four dengue viruses (DENV-1 to 4). Each of these viruses is capable of causing nonspecific febrile illnesses, classic dengue fever and dengue haemorrhagic fever (Gubler 1998). As a result, dengue is often difficult to diagnose clinically, especially because peak dengue season often coincides with that of other common febrile illnesses in tropical regions (Chacon et al. 2015). In April 2009, a new virus, inﬂuenza A/H1N1/pandemic (FluA/H1N1/09pdm), caused a severe outbreak in Mexico. The virus quickly spread throughout the world, and in June 2009, the World Health Organization declared a pandemic (WHO 2010). In Brazil, the first laboratory confirmed case of FluA/H1N1/09pdm was in July 2009 (Pires Neto et al. 2013). The state of Ceará, in Northeast Brazil, is a dengue endemic area. In this state, the virus influenza A(H1N1)pdm09 has circulated since 2009, and through the first half of 2012, 11 deaths caused by the virus were confirmed (Pires Neto et al. 2013). The influenza and dengue seasons in Ceará overlap, which led to diagnostic difficulties. We report four cases of laboratory-confirmed coinfection of deadly influenza A(H1N1)pdm09 with DENV, which occurred during the dengue and influenza season in 2012 and 2013 in Ceará.

All subjects enrolled in this study had no definite diagnosis at the time of death and were sent to Dr Rocha Furtado Death Verification Service (DVS) of the State Health Secretariat of Ceará, Brazil, for diagnosis. Pathologists at the DVS suspected a diagnosis of dengue or influenza after reviewing clinical records, during an interview with relatives, or during autopsy. The criteria for suspicion of dengue were (i) a recent report of fever (maximum seven days) with no apparent bacterial infection; (ii) the presence of a rash; (iii) the presence of cavity effusion and/or bleeding (Cavalcanti et al. 2016). For influenza, the criteria used were a sudden onset of fever and sore throat reports, cough or dyspnea (Chacon et al. 2015). The bodies were necropsied after family consent, and samples of tissues and secretions were collected and sent to the Central Public Health Laboratory for testing for dengue and influenza to clarify the cause of death.

All lungs were processed, and real-time reverse transcriptase polymerase chain reaction (RT-PCR) was performed for influenza A(H1N1)pdm09. The real-time RT-PCR was performed in accordance with the protocols established by the WHO/CDC (2009) for the detection and characterisation of influenza A(H1N1)pdm09, including a panel of oligonucleotide primers and dual-labelled hydrolysis (Taqman®). A real-time PCR System 7500 thermocycler and its corresponding software (Applied Biosystems, Foster City, CA, USA) were used.

Liver fragments were used for the diagnosis of dengue. Serum and cerebrospinal fluid (CSF) were also used when available. One liver fragment was fixed in 10% formalin for immunohistochemistry (IHC), which was performed in Instituto Evandro Chagas. The IHC technique to determine the presence of dengue antigen in formalin-fixed, paraffin-embedded liver tissue samples used alkaline phosphatase as the enzyme labelling system, with a red chromogen that contrasted with the tissue pigments and mouse polyclonal antibody from ascitic fluid against dengue, using negative and positive controls, as described previously (Hall et al. 1991). Another liver fragment was processed by real-time RT-PCR for dengue in accordance with protocols established by the CDC (2013). Antigen detection by NS1 and IgM antibodies in the serum and CSF was performed using the Panbio kit, as described by Araújo et al. (2011).

The first case was a 30-year-old woman who lived in the countryside and worked as a housekeeper. She first presented with symptoms, such as haemoptysis and dyspnea, on March 27, 2012. Three days later, she went to the hospital with fever, myalgia, cough and headache. She was admitted and evolved to severe respiratory failure. She had a platelet count of 89.000/mm^3^, and no comorbidity was reported. She evolved to death on the same day, and the diagnostic hypotheses were severe respiratory failure, pneumonia, tuberculosis and flu (Table I). At the hospital, the doctor did not reach a conclusion about the cause of death, and the body was sent to DVS for diagnosis. The necropsy findings were peripheral cyanosis, bilateral pleural effusion, pericardial effusion, heart enlargement, hepatomegaly and congested kidneys. Microscopy showed cerebral edema, congestive heart and edema, hypertrophy of myocardial fibres and dilation of the chambers. The lung hyaline membranes were thick and had interstitial pneumonitis and intra-alveolar edema. The kidneys had acute tubular necrosis. The liver showed cholestasis. Fragments of viscera with and without formaldehyde were sent to the laboratory for analysis. The lung fragments were positive for influenza A(H1N1)pdm09 virus by real-time RT-PCR, and the liver fragments were positive for dengue viruses (DENV) by immunohistochemistry. Pathologist’s conclusion: shock; pneumonitis associated with FluA/H1N1/pdm09 virus. Associated condition: dengue.

**TABLE I:** Clinical epidemiological features and pathologist’s conclusion of four fatal influenza/dengue co-infection cases

Case	Ager/Gender	Diagnostic hypothesis	Symptoms/Sigs	Underlying conditions	Days between onset of symptoms and death (nº)	Pathologist’s conclusion
1	30/F	Severe respiratory failure; pneumonia; tuberculosis; influenza	Hemoptysis; fever; myalgia; cough; headache; dyspnea; platelet count 89.000/mm^3^	None	3	Shock; pneumonitis associated with pandemic Flu. Associated condition: dengue
2	30/M	Dengue and influenza	Fever; chest pain; headache; retro-orbital pain; dry cough; diarrhea; vomiting; dyspnea; platelet count 151.000/mm^3^ ; aspartate aminotransferase 541IU/L; alanine aminotransferase 90IU/L	Alcoholic	4	Septic shock; broncopneumonia caused by Streptococcus pyogenes; pandemic Flu. Associated condition: dengue
3	31/F	Influenza and pneumonia	Fever; dry cough; dyspnea	Hypertension and obesity	10	Acute respiratory failure; pandemic flu. Associated condition: dengue
4	54/M	Influenza and pneumonia	Fever; cough; dyspnea	Hypertension	7	Diffuse alveolar damage and mild acute pneumonia; pandemic Flu. Associated condition: dengue and hypertension.

The second case was a 30-year-old man who lived in the capital city and worked as an agricultural worker. He was an alcoholic and presented with fever, chest pain, headache, retro-orbital pain, dry cough, diarrhoea, vomiting, and progressive worsening of dyspnea on April 3, 2012. The next day, he began to use multiple symptomatic drugs (dipyrone, paracetamol). On April 7, he was received at the emergency hospital dehydrated, with cold skin and dyspnea evolving to tonic-clonic convulsions and cardiorespiratory arrest. He was taken to the resuscitation room for cardiopulmonary resuscitation, which was unsuccessful. Analyses carried out prior to death were: creatinine, 3.79 mg/dL; potassium, 3.56 nmol/L; sodium, 131.5 nmol/L, aspartate aminotransferase, 541 IU/L; alanine aminotransferase, 90 IU/L; platelet count, 151,000/mm^3^. The diagnostic hypothesis was dengue and influenza (Table I). To clarify the cause of death, the body was sent to the DVS. The necropsy findings were the following: incarcerated inguinal hernia; pulmonary edema; cardiomalacia; left ventricular hypertrophy; pleural and pericardial sero-haemorrhagic effusion (200 mL); discreet hepatomegaly and congested kidneys. Microscopic examination showed severe acute bronchopneumonia; intra-alveolar edema; hyaline membranes; interstitial myocardial edema; moderate hepatic steatosis in large drops in the midzone; severe sinus congestion; intensely congested spleen; numerous bacterial and hyaline cylinders; moderate glomerulosclerosis. Fragments of viscera with and without formaldehyde and effusions were sent to the laboratory for analysis. The lung fragments were positive for influenza A(H1N1)pdm09 virus by real-time RT-PCR, and the liver fragments were positive for dengue by IHC. The culture of effusions isolated *Streptococcus* pyogenes (Lancefield group A). Gram staining showed gram-positive cocci in pairs and chains. Pathologist’s conclusion: septic shock; acute bronchopneumonia; infection by *Streptococcus* pyogenes; influenza A(H1N1)pdm09 virus. Associated conditions: dengue, chronic alcoholism.

The third case was a 31-year-old woman who lived in the countryside, worked as an agricultural worker, and reported having respiratory symptoms for ten days. She was admitted to the hospital with a dry cough, fever, and dyspnea, and her condition evolved with worsening of symptoms. She was admitted to the hospital with severe respiratory insufficiency and evolved to cardiorespiratory arrest. When she was in the hospital, she was given antibiotics. She died on April 26, 2012. She presented comorbidities, hypertension and obesity. The diagnostic hypotheses at death were influenza and pneumonia (Table I). The body was sent to DVS in order to clarify the cause of the death. The necropsy findings were as follows: discreet bilateral pleural and pericardial serous effusion; edema and more pronounced pulmonary congestion on the bases; dilation of the cardiac chambers; acute infection in the spleen; edema and pulmonary congestion more pronounced on the bases; fibrosis in the kidneys. Microscopic examination showed as follows: acute necrotising bronchitis and bronchiolitis; heavily exudative diffuse interstitial pneumonitis; thick hyaline membranes covering the lining of alveoli and bronchiolar surfaces; significant intra-alveolar edema; the presence of giant cells in the alveolar lining and alveolar septum, some with irregular nuclei blurred chromatin; a large amount of intra-alveolar cell debris; acute bronchopneumonitis in small, non-confluent foci; pulmonary embolism; myocardial interstitial edema; acute hepatitis; moderate hyperplasia of Kupffer cells; some hyaline corpuscles and focal necrosis of hepatocytes; diffuse hepatic steatosis in small drops predominating in the midzone; moderate congestion of the spleen; extensive acute tubular necrosis; and nodular glomerulus sclerosis. The lung fragments were positive for Influenza A(H1N1)pdm09 virus by real-time RT-PCR, and the liver fragments were positive for dengue by IHC. Pathologist’s conclusion: acute respiratory failure; influenza A(H1N1)pdm09 virus. Associated conditions: dengue, hypertension and obesity.

The fourth case was a 54-year-old man who lived in the countryside and worked as a cooling technician. One week before his death, he presented fever, cough, and dyspnea and was diagnosed with pneumonia. He was admitted to the hospital with progressive worsening and respiratory failure. He was intubated and evolved into cardiorespiratory arrest on May 16, 2013. He was treated with antibiotics for pneumonia, but without improvement. He presented comorbidities, including hypertension. The diagnostic hypothesis was influenza and pneumonia (Table I). The body was sent to DVS in order to clarify the cause of death. The necropsy findings were as follows: cardiomegaly with left ventricular hypertrophy; pulmonary edema; congested liver; kidneys and other organs without macroscopic changes. Microscopy showed mild interstitial fibrosis of the kidneys; centrilobular congestion with necrotic foci in the liver; lungs with hyaline membranes and discreet pneumonic exudation; hypertrophy of the cardiac muscle. The lung fragments were positive for influenza A(H1N1)pdm09 virus by real-time RT-PCR; the liver fragments were positive for dengue by IHC, and real-time RT-PCR detected DENV-4; the sera and CSF were positive for IgM antibody and NS1 antigen. Pathologist’s conclusion: death by diffuse alveolar damage and mild acute pneumonia, hypertrophic cardiomyopathy and hypertension; FluA/H1N1/pdm09 virus. Associated conditions: dengue and hypertension.

The cases reported here showed some similarities: all were young adults who presented with dengue coinfection. In England and in São Paulo, Brazil, fatal influenza A(H1N1)pdm09 was mainly observed in young adults, unlike what is observed for seasonal influenza (Pebody et al. 2010, Ribeiro et al. 2015). Notably, three of the cases had underlying conditions, such as obesity (Table I), which was considered a risk condition for the severity of the disease (Morgan et al. 2010, Kwong et al. 2011). The clinical syndromes produced by influenza and infection with DENV can be mimicked by each other, making it difficult to diagnose: the former may cause a febrile illness, headache, myalgia and haemorrhagic manifestations in atypical cases, suggesting dengue infection; the latter may cause pleural effusion, pneumonitis, pulmonary haemorrhage and acute respiratory failure, resembling influenza infection. The possibility of asymptomatic infection with DENV also should be considered (Gupta et al. 2009, Chudasama et al. 2011, Wiwanitkit & Wiwanitkit 2013). In relation to necropsy and the histopathological features of the cases, some findings were also common to both infections (Table II). Patients sought hospital care after the worsening of symptoms, which was perhaps too late because they died within hours after they were admitted. It is known that the management of this coinfection requires aggressive early treatment (Behera et al. 2015); however, although the suspected diagnosis was influenza, no patient was treated with oseltamivir. Only one patient had the diagnostic hypothesis of dengue and influenza, although all patients were confirmed by the pathologist to have influenza and dengue coinfection. Previous studies have described cases of coinfection with influenza A(H1N1)pdm09 and dengue virus (Pérez et al. 2010, Rodríguez et al. 2010). In dengue-endemic countries, providers should consider the possibility of influenza and dengue coinfection earlier in the process in order to avoid late diagnosis and fatal outcomes. Further studies of necropsies are required to recognise pathological findings that make it possible to draw a clinical pathology profile for dengue and influenza coinfection.

**TABLE II:** Necropsy and histopathologic findings in four fatal cases of FluA/H1N1/pdm09 and dengue co-infection in different available tissues

Organ	Case 1	Case 2	Case 3	Case 4
Brain	Cerebral edema	-	-	-
Lung	Bilateral pleural effusions; interstitial pneumonitis; intra-alveolar edema	Pleural sero-haemorrhagic effusion; pulmonary edema; bronchopneumonia	Bilateral pleural effusion; edema; congestion; bronchitis; bronchiolitis; interstitial pneumonitis	Edema; hyaline membranes and pneumonia exudation
Heart	Congestion; edema pericardial e ffusion heart in largement	Cardiomalacia; left ventricular hypertrophy; edema; interstitial myocarditis	Pericardial effusion; edema	Cardiomegaly; left ventricular hypertrophy
Liver	Cholestasis; hepatomegaly	Hepatomegaly and steatosis	Acute hepatitis; necrosis	Congestion and necrosis
Spleen	-	Congestion	Acute infections spleen; congestion	-
Kidney	Congested kidneys and acute tubular necrosis	Congested kidneys; bacterial and hyaline cylinders; glomerulosclerosis	Fibrosis and necrosis	Interstitial fibrosis
